# Regulation of TDP-43 phosphorylation in aging and disease

**DOI:** 10.1007/s11357-021-00383-5

**Published:** 2021-05-25

**Authors:** Randall J. Eck, Brian C. Kraemer, Nicole F. Liachko

**Affiliations:** 1 Neuroscience Graduate Program, University of Washington, Seattle, WA 98195 USA; 2 Geriatric Research Education and Clinical Center, Seattle Veterans Affairs Puget Sound Health Care System, 1660 South Columbian Way, Seattle, WA 98108 USA; 3 Division of Gerontology and Geriatric Medicine, Department of Medicine, University of Washington, Seattle, WA 98104 USA; 4 Department of Psychiatry and Behavioral Sciences, University of Washington, Seattle, WA 98195 USA; 5 Department of Laboratory Medicine & Pathology, University of Washington, Seattle, WA 98104 USA

**Keywords:** TDP-43, Amyotrophic lateral sclerosis (ALS), Frontotemporal lobar degeneration (FTLD), Phosphorylation, Kinases, Phosphatases

## Abstract

Insoluble inclusions of phosphorylated TDP-43 occur in disease-affected neurons of most patients with amyotrophic lateral sclerosis (ALS) and about half of patients with frontotemporal lobar degeneration (FTLD-TDP). Phosphorylated TDP-43 potentiates a number of neurotoxic effects including reduced liquid–liquid phase separation dynamicity, changes in splicing, cytoplasmic mislocalization, and aggregation. Accumulating evidence suggests a balance of kinase and phosphatase activities control TDP-43 phosphorylation. Dysregulation of these processes may lead to an increase in phosphorylated TDP-43, ultimately contributing to neurotoxicity and neurodegeneration in disease. Here we summarize the evolving understanding of major regulators of TDP-43 phosphorylation as well as downstream consequences of their activities. Interventions restoring kinase and phosphatase balance may be a generalizable therapeutic strategy for all TDP-43 proteinopathies including ALS and FTLD-TDP.

## Introduction

The TAR DNA-binding protein (TDP-43), encoded by the highly conserved *TARDBP* gene, is a nucleic acid binding protein originally identified as a transcriptional repressor of the human immunodeficiency virus type 1 (HIV-1) trans-activator response (TAR) long terminal repeats [1]. Since then, TDP-43 has been shown to participate in a variety of critical RNA metabolism activities including transcriptional regulation, pre-mRNA splicing of most transcripts, alternative splice site selection, mRNA stability and transport, and microRNA biogenesis (reviewed in [2]). The discovery that TDP-43 comprised the major protein component of ubiquitinated inclusions in both amyotrophic lateral sclerosis (ALS) and frontotemporal lobar degeneration (FTLD-TDP) began serious investigation into the role of TDP-43 in neurodegenerative diseases of aging [3–5]. Mutations identified within the *TARDBP* gene cause some familial cases of ALS, indicating pathological TDP-43 is not only a hallmark of disease, but also a driver of disease [6].

**Fig. 1:**
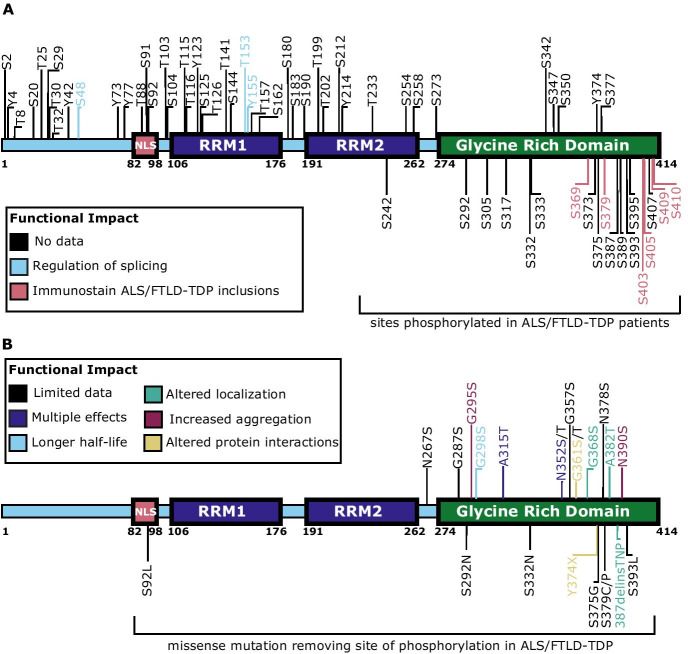
TDP-43 protein domains and phosphorylation sites. **A** TDP-43 is a 414 amino acid nucleic acid binding protein involved in RNA metabolism activities including transcriptional regulation, pre-mRNA splicing, mRNA stability and transport, and microRNA biogenesis. TDP-43 contains an N-terminus with a nuclear localization signal (NLS), two RNA recognition motifs (RRM1 and RRM2), and a C-terminus glycine-rich low-complexity domain. TDP-43 has 64 potential phosphorylation sites: 41 serine (Ser), 15 threonine (Thr), and 8 tyrosine (Tyr) residues. A subset of these sites (shown below the TDP-43 diagram) have been identified phosphorylated in ALS and FTLD-TDP patients. Many of TDP-43’s phosphorylation sites have not been fully characterized although in vivo and in vitro studies have used immunostaining to demonstrate their presence in TDP-43-positive inclusions (red text), or have linked specific sites to the regulation of TDP-43 splicing activity (blue text) (see inset box). **B** A subset of disease-causing TDP-43 missense mutations delete (shown below the TDP-43 diagram) or introduce (shown above the TDP-43 diagram) phosphorylation sites. Some of these mutations’ impact on TDP-43 pathology have been characterized, as represented by their font color (see inset box and color coding of text), including increased TDP-43 cytoplasmic mislocalization, altered protein–protein interactions, increased aggregation, and/or increased protein half-life. While additional characterization is needed, these mutations suggest a key role for the regulation of phosphorylation in TDP-43 function and disease

Pathological TDP-43 accumulates in the disease-affected neurons of ~ 97% of ALS patients and ~ 50% of FLTD patients [7–9]. This typically manifests as cytoplasmic deposition of C-terminally phosphorylated TDP-43 (Fig. 1A). The best characterized of these phosphorylation events occurs at serines 409 and 410 (S409/410), which are consistently phosphorylated in disease. In addition to being a robust and consistent pathological marker for TDP-43-positive inclusions [10], phosphorylated TDP-43 manifests in other neurodegenerative disease and aging [11]. TDP-43 pathology has been strongly associated with hippocampal sclerosis [12, 13] and accumulates in a subset of Alzheimer’s disease cases, with dementia severity correlating dose dependently with phosphorylated TDP-43 deposition [14, 15]. Furthermore, TDP-43 is increasingly recognized as a secondary pathology of other neurodegenerative disorders including Lewy body–related diseases and Huntington’s disease [16, 17].

TDP-43 pathology can accumulate in non-demented aged individuals [18], although a recently classified TDP-43 proteinopathy, limbic predominant age-related TDP-43 proteinopathy (LATE), mimics Alzheimer’s disease clinically in patients of advanced age. Compared to Alzheimer’s disease, LATE exhibits a restricted distribution of TDP-43 pathology frequently associated with hippocampal sclerosis and does not display the plaques and tangles defining Alzheimer’s disease [19]. Phosphorylated TDP-43 can also become deposited due to environmental insults as occurs in chronic traumatic encephalopathy (CTE) following brain injury [20]. The genetic or environmental causes of TDP-43 pathological deposition are unknown in the majority of patients with ALS, FTLD-TDP, Alzheimer’s disease, Lewy body disease, Huntington’s disease, or LATE. It remains possible that diverse mechanisms lead to the pathognomonic phenotypes observed in these different neurodegenerative diseases. Taken together, these observations support a role for TDP-43 in pathological aging and suggest pathological TDP-43 has a widespread impact on the cognitive decline associated with aging (reviewed in [21]).

## TDP-43 structure and function

TDP-43 contains protein domains consistent with its known functions, including an N-terminus with a nuclear localization signal (NLS), two RNA recognition motifs (RRM1 and RRM2), and a C-terminus glycine-rich low-complexity domain (Fig. 1) [22]. RRM2 also contains a putative nuclear export signal, although it is not required or sufficient to mediate nuclear egress [23]. The N-terminus of TDP-43 functions in dimerization and can bind single-stranded DNA [24–26]. Both RRM1 and RRM2 are required for TDP-43 to bind UG-rich RNA or TG-rich DNA sequences, although RRM2 appears to play a supporting role to RRM1 [27–30]. Mutations that disrupt TDP-43 RNA binding may contribute to pathogenic mislocalization and aggregation in ALS and FTLD-TDP [31]. While primarily localized in the nucleus, TDP-43 can freely shuttle between the nucleus and cytoplasm and has also been observed in neuronal mitochondria in ALS and FTLD-TDP [32, 33]. During cellular stress, TDP-43 localizes to the cytoplasm and can associate with stress granules through its RRM and C-terminus domains [34]. TDP-43 undergoes liquid–liquid phase separation, mediated by its C-terminus low-complexity domain [35, 36]. Deletion of this region prevents aggregation [37], and ALS-associated mutations in the C-terminus disrupt phase separation and promote aggregation [36, 38, 39]. Additionally, TDP-43 interactions with other proteins including ubiquilin-2 are mediated by the C-terminus domain [40–42]. Finally, through these various protein domains (Fig. 1), the cellular activities of TDP-43 are regulated by a combination of post-translational modifications, subcellular localization, and specific interactions with co-factors.

## TDP-43 phosphorylation sites in disease

In ALS and FTLD-TDP, aggregated TDP-43 exhibits several post-translational modifications including ubiquitination, acetylation, SUMOylation, and phosphorylation [3, 4, 43, 44]. Of these modifications, abnormal phosphorylation of TDP-43 is a highly consistent marker of disease and is used diagnostically to identify TDP-43-positive protein inclusions in brain and spinal cord [5, 10, 45]. TDP-43 has 64 potential phosphorylation sites: 41 serine (Ser), 15 threonine (Thr), and 8 tyrosine (Tyr) residues (Fig. 1A). A subset of these sites become phosphorylated in vivo, a smaller group have been observed by IHC in ALS and FTLD-TDP patients, and fewer have been functionally characterized. Mass spectrometry of insoluble TDP-43 from the brain tissue of two ALS patients showed TDP-43 was phosphorylated at 17 sites, 16 of which reside in the glycine-rich C-terminus domain [46]. In addition to these 17 sites, two other sites — S369 and S410 — are known to be phosphorylated in ALS and FTLD-TDP patients [5, 10, 47, 48]. Of these 19 sites, phosphorylation at S369, S379, S403/404, and S409/410 consistently occurs in the context of disease suggesting a potential pathological role (Fig. 1A). The distribution of the remaining 13 sites in disease has not yet been corroborated with IHC or other independent means, due to a lack of phosphorylation site-specific antibodies. Some of the identified phosphorylation events may represent the consequences of priming by disease-linked sites.

At least twenty disease-linked missense mutations have been identified that introduce or delete phosphorylation sites potentially altering TDP-43’s localization, aggregation, half-life, or protein–protein interactions (Fig. 1B) (reviewed in [6]). These mutations may also promote phosphorylation at nearby phospho-sites. However, characterization of the biological impact of these changes is ongoing. It remains unknown whether TDP-43 phosphorylation status changes with age to predict disease onset, or whether there are disease-specific patterns of TDP-43 phosphorylation. However, derangement of kinase or phosphatase activities with aging or disease may promote increased TDP-43 phosphorylation leading to dysfunction and eventual neurodegeneration. Given the ubiquitous presence of TDP-43 phosphorylation in disease, understanding the functional impact of specific phosphorylation sites is likely to provide important insights into disease mechanisms.

## Regulation of TDP-43 phosphorylation

**Fig. 2:**
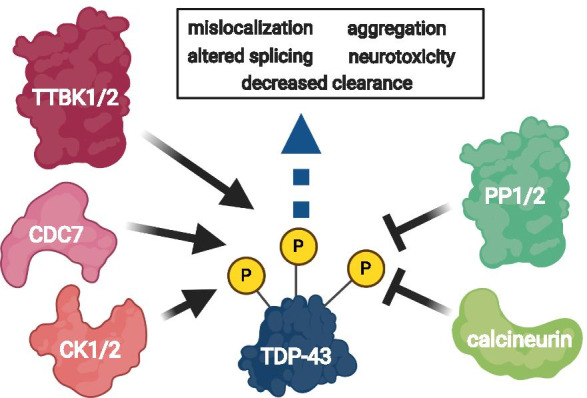
Regulation and consequences of TDP-43 phosphorylation. TDP-43 phosphorylation is linked to deleterious functional changes including altered splicing activity, cellular mislocalization, decreased turnover, changes in solubility, and increased neurotoxicity. Five kinases have been shown to directly phosphorylate TDP-43: casein kinases 1 and 2 (CK1 and CK2), cell division cycle 7 (CDC7), and tau tubulin kinases 1 and 2 (TTBK1 and TTBK2). The phosphatases PP1, PP2, and calcineurin have been found to interact with and dephosphorylate TDP-43. Kinase and phosphatase activities regulate TDP-43 phosphorylation in what is likely to be dynamic cellular process with potential impacts on TDP-43 activities, interaction partners, and localization. Interventions restoring kinase and phosphatase balance may be a generalizable therapeutic strategy for all TDP-43 proteinopathies including ALS and FTLD-TDP

While phosphorylation of TDP-43 could represent an aberrant cellular process during disease, several lines of evidence suggest this is a regulated event. To date, five kinases have been shown to directly phosphorylate TDP-43: casein kinases 1 and 2 (CK1 and CK2), cell division cycle 7 (CDC7), and tau tubulin kinases 1 and 2 (TTBK1 and TTBK2) (Fig. 2) [5, 49–51]. In vitro*,* the kinases CK1 and CK2 promote TDP-43 polymerization into electron microscopy-evaluated structures reminiscent of those from FTLD-TDP [52], and all five kinases can phosphorylate purified TDP-43, with CK1 phosphorylating TDP-43 at 29 known sites [5, 49–51]. These kinases actively target TDP-43 in vivo, as modulation of kinase activity alters TDP-43 phosphorylation. For example, inhibitors of the kinases CK1 and CDC7 have been shown to prevent accumulation of phosphorylated TDP-43 and protect against neuron loss, while overexpression of CDC7, TTBK1, or TTBK2 promotes TDP-43 phosphorylation [49, 53–57]. CDC7, TTBK1, and TTBK2 also co-localize with aggregated, phosphorylated TDP-43 in ALS and FTLD-TDP [49, 50]. Additionally, casein kinase 1ε (*CSNK1E*) mRNA levels correlate with phosphorylated TDP-43 in ALS patient tissue and overexpression of CSNK1E in iPSC-derived motor neuron lines promotes TDP-43 cytoplasmic accumulation [58]. Finally, although not shown to directly phosphorylate TDP-43, the kinase MEK promotes increased dual phosphorylation at T153/Y155 in the TDP-43 RRM1 domain and nucleolar localization of TDP-43 during the heat shock response in SH-SY5Y cells [59]. It remains unknown whether TDP-43 kinases act in a defined temporal sequence, or whether their activities are coordinated with C-terminal truncation of TDP-43. The full phosphorylation profiles and site-preferences of these kinases on TDP-43 have also yet to be fully characterized. It is clear, though, that phosphorylation of TDP-43 by these kinases modifies pathology in model systems, and likely serves normal endogenous roles in regulating protein activity in conjunction with phosphatases.

Phosphatases catalyze the reverse reaction, removing phosphate groups from modified tyrosine, serine, and threonine residues. The phosphatases PP1, PP2, and calcineurin have been found to dephosphorylate TDP-43 (Fig. 2). All three phosphatases can physically interact with TDP-43, as PP1 and PP2 co-immunoprecipitated with TDP-43 from HEK-293FT cells, while calcineurin interacted with the C-terminus of TDP-43 in a yeast-2-hybrid assay [60, 61]. In addition, calcineurin and TDP-43 co-immunoprecipitated from human brain lysate [62]. PP1 can dephosphorylate purified human TDP-43 at pathological sites S379, S403, S404, S409, and S410 in vitro, while calcineurin has been shown to dephosphorylate TDP-43 at S409 and S410 [60, 61]. In mammalian cell culture, pharmacological inhibition of PP1 using the drug Calyculin A, or inhibition of calcineurin using the drugs FK506 or Cyclosporin A, drives accumulation of phosphorylated TDP-43 [60, 61]. In TDP-43-expressing transgenic *C. elegans*, genetic loss of the sole calcineurin A homolog, *tax-6*, dramatically worsens motor phenotypes, phosphorylated TDP-43 accumulation, and neurodegeneration. Calcineurin and phosphorylated TDP-43-positive lesions co-localize in cortical neurons from patients with FTLD-TDP, and in spinal cord motor neurons from patients with ALS, suggesting calcineurin may be recruited to dephosphorylate TDP-43 in human disease [61]. Calcineurin activity is decreased in both sporadic and familial ALS patients, making calcineurin dysregulation a possible contributor to disease [63, 64]. Taken together, kinase and phosphatase activities regulate TDP-43 phosphorylation in what is likely to be a dynamic cellular process influenced by aging or disease and with consequences for TDP-43 activities, interaction partners, and localization.

## Consequences of TDP-43 phosphorylation

While there has not yet been an exhaustive survey of the functional consequences of TDP-43 phosphorylation at all possible phospho-sites, several sites have been examined in detail in model systems.

### Regulation of splicing activity

In vitro and in vivo models have found phosphorylation at S48 and T153/Y155 can alter TDP-43’s splicing activity [59, 65]. A phosphomimetic substitution at S48 (S48E) reduces TDP-43’s alternative splicing repressor activity, resulting in a 40% reduction in the inclusion of exon 9 in the mRNA of cystic fibrosis transmembrane conductance regulator (CFTR) of HeLa cells [65]. Mutations T153E/Y155E, a likely dual phosphomimic, similarly reduce TDP-43 splicing activity, decreasing the inclusion of CFTR exon 9 by 30% compared to wild type. T153E/Y155E also decreases TDP-43 A(GU)_6_ RNA binding affinity fivefold, while not altering solubility or degradation [59], further suggesting a specific role for phosphorylation in regulating splicing activity.

### Subcellular localization

In addition to altered splicing activity, TDP-43 phosphorylation at T153/Y155 in SH-SY5Y cells regulates TDP-43 recruitment to nucleoli [59]. TDP-43 can also accumulate in mitochondria, and a phosphomimetic G298D mutation within one of TDP-43’s mitochondrial localization signals increases TDP-43 mitochondrial localization in HEK-293 cells compared to wild type [32]. TDP-43 is predominantly located in the nucleus; however, in a model of intercranial hemorrhage (ICH) in rats and rat primary neurons, TDP-43 translocates to the cytoplasm following ICH. Overexpressed, non-phosphorylatable TDP-43 mutants S409A/S410A remain in the nucleus following ICH, suggesting a role for phosphorylation in TDP-43 cytoplasmic localization. The authors also found TDP-43 S409A/S410A mutations correlated with a reduction in markers of autophagosome-lysosome fusion and increased mTOR and dynactin1 levels following ICH, potentially due to the maintenance of nuclear localized TDP-43 [66]. Following ER stress, the kinase CK1 promotes TDP-43 cytoplasmic accumulation in NSC-34 cells [67]. Expression of phosphomimetic mutations S375E and S387E/S389E/S393E/S395E in TDP-43’s low-complexity domain also significantly increases TDP-43 cytoplasmic localization compared to wild type in HeLa cells [68]. In other models, cytoplasmic accumulation of TDP-43 is sufficient to induce aggregation and phosphorylation. For example, the deletion of TDP-43’s NLS induces TDP-43 aggregation in phase separated droplets in SH-SY5Y cells. Long-lived TDP-43 phase separated droplets also recruit phosphorylated TDP-43 [69]. In transgenic mice, expression of human TDP-43 with a defective NLS results in accumulation of cytoplasmic phosphorylated insoluble TDP-43, accompanied by motor impairment, neurodegeneration, and rapid death [70].

### Solubility and aggregate formation

Independent of localization, phosphorylation of TDP-43 has been linked to changes in solubility and aggregate formation. A phosphomimetic substitution at S48 (S48E) dramatically reduces the ability of TDP-43 to polymerize. Furthermore, S48E slows TDP-43 phase separation and results in more soluble phase separated TDP-43 nuclear assembles [65]. A recent study also found this mutation disrupted the formation of anisosomes, distinct phase separated nuclear assemblies formed when TDP-43 polymerizes following an inhibition of RNA binding [71]. Beyond phase separation, phosphorylation at S409/410 in the C-terminus of TDP-43 fragments increases their resistance to proteasomal degradation [72]. Phosphorylation of TDP-43 at S403/404 in HeLa cells by CK1, CK2, TTBK1, or TTBK2 inhibits calpain-dependent cleavage of TDP-43 compared to non-phosphorylated TDP-43 in vitro [73]. Previous research found calpain cleaved TDP-43 fragments are more soluble and suggests preventing calpain-dependent digestion could increase TDP-43 aggregation propensity [74]. In another experiment, the co-expression of constitutively active mutant CSNK1E and TDP-43 in SH-SY5Y cells resulted in dual phosphorylation of TDP-43 at S393/395 and increased TDP-43 aggregation. Mutation of S393/395 to the non-phosphorylatable S393A/S395A reduces the levels of TDP-43 phosphorylation at other sites including S409/410, as well as the total amount of accumulated TDP-43 in the sarksoyl-insoluble fraction of cells [75]. Another study suggests phosphorylation at S409/410 is a secondary event in seed-dependent TDP-43 aggregation [76]. In N2A or COS-7 cells expressing 15 kDa and 25 kDa C-terminus fragments of TDP-43, phosphorylation of S409/410 is significantly more common in insoluble fragments further supporting a role for S409/410 phosphorylation in reducing solubility [76].

However, not all studies suggest phosphorylation promotes aggregation. In HEK-293 cells, TDP-43 15 kDa and 25 kDa fragments with phosphomimetic mutations S409D/S410D show 60% less aggregation compared to wild type fragments suggesting a possible compensatory role for TDP-43 phosphorylation. Non-phosphorylatable mutations S409A/S410A also reduce aggregation, but only by 15% [77]. Likewise, expressing TDP-43 C-terminus fragments with S403A/S404A or S409A/S410A non-phosphorylatable mutations in *Drosophila* does not alter TDP-43 aggregation. Various combinations of phosphomimetic mutations S379E, S403E, S404E, S409E, and S410E, or expression of the kinase CK2α, are able to reduce the number of aggregates in cells. In these studies, S409/410 phosphorylation occurs after aggregation suggesting TDP-43 phosphorylation may be compensatory, delaying the formation of larger aggregates by electrostatic repulsion at the expense of decreasing TDP-43 turnover [78].

### Timing of phosphorylation

While the timing and subcellular localization of TDP-43 phosphorylation in disease remains largely unexplored, two recent studies support S409/410 phosphorylation occurring after aggregate formation. To drive aggregation, an optogenetic TDP-43 construct that undergoes oligomerization after blue light exposure (optoTDP43) was expressed in HEK-293 cells or ReNcell cortical neuron cultures. After induction, optoTDP43 forms stable neurotoxic inclusions, a subset of which are phosphorylated [79]. Another study induced the formation of stress granules using optogenetic multimerization of the stress granule scaffold protein G3BP1. TDP-43 is recruited to these OptoGranules and became immunopositive for S409/410 phosphorylation after recruitment [80]. During transient stress, TDP-43 recruitment to stress granules inhibits TDP-43 phosphorylation at S409/410. It is possible the stress granule environment excludes stress-responsive kinases, altering TDP-43 interactions with kinases and phosphatases and delaying cytoplasmic accumulation of TDP-43 [81].

### Neurotoxicity

In *C. elegans* models, neuronal expression of human TDP-43 with fALS mutations G290A or M337V kills motor neurons. Prevention of S409/410 phosphorylation with non-phosphorylatable mutations S409A/S410A improves locomotor dysfunction [82]. Similarly, co-expression of mutant human TDP-43 Q331K with *doubletime*, the fly homolog of CSNK1E, in the *Drosophila* eye increases S409/410 phosphorylation and the formation of toxic oligomeric species. Cytotoxicity of TDP-43 oligomers is also enhanced in SY5Y cells when treated with a recombinant rat CK1 [83].

## Summary

Taken together, the referenced studies demonstrate that TDP-43 phosphorylation influences normal TDP-43 functions including RNA binding, alternative splicing, localization, phase separation, aggregation, and clearance. Yet, many questions remain with regard to the sub-cellular localization, timing in disease progression, potential for additional phosphorylation priming, and full functional impact of site-specific phosphorylation. For example, phospho-specific antibodies raised against TDP-43 phosphorylated at S369 and S379 immunostain insoluble cytoplasmic inclusions in ALS/FTLD-TDP patient brain tissue, but the functional impact of phosphorylation at these sites on TDP-43 is unknown. Disease-causing missense mutations introducing or deleting TDP-43 phosphorylation sites also point to important roles for phosphorylation in health and disease (Fig. 1B). It remains unknown whether changes in the pattern or degree of TDP-43 phosphorylation presage development of neuronal dysfunction or neurodegeneration, or whether normal aging affects regulation of TDP-43 phosphorylation. Nevertheless, this work represents an important start to understanding the variety of regulatory effects of TDP-43 phosphorylation.

The kinases and phosphatases that regulate TDP-43 phosphorylation are possible therapeutic targets in TDP-43 proteinopathies. However, a single primary TDP-43 kinase has not been determined in neurons; given possible overlap or redundancy in TDP-43 kinases’ activities, effective treatments may require multiple small molecule inhibitors targeting several TDP-43 kinases to fully prevent TDP-43 phosphorylation. Similarly, phosphatase activation could be used to drive clearance of phosphorylated TDP-43, although given the many known targets of calcineurin, PP1, and PP2, this strategy would need to be carefully evaluated to screen for potential adverse effects. Another strategy could be targeted clearance of phosphorylated TDP-43, potentially using an immunotherapy approach, or by enhancing endogenous cellular clearance mechanisms such as autophagy. Given the interplay among TDP-43 phosphorylation, aggregation, and cellular localization, treatments focused on disaggregation or restoration of nuclear localization could reduce TDP-43 phosphorylation as a downstream consequence, with potential neuroprotective benefits. There is an urgent need for effective therapies for TDP-43 proteinopathies; modifying TDP-43 phosphorylation holds both promise and challenge for ALS and FTLD-TDP.
